# The Physiological Effects of Air Pollution: Particulate Matter, Physiology and Disease

**DOI:** 10.3389/fpubh.2022.882569

**Published:** 2022-07-14

**Authors:** Jack T. Pryor, Lachlan O. Cowley, Stephanie E. Simonds

**Affiliations:** ^1^Metabolism, Diabetes and Obesity Programme, Monash Biomedicine Discovery Institute, Monash University, Clayton, VIC, Australia; ^2^Woodrudge LTD, London, United Kingdom

**Keywords:** PM2.5, disease, physiology, particulate matter, air pollution

## Abstract

Nine out of 10 people breathe air that does not meet World Health Organization pollution limits. Air pollutants include gasses and particulate matter and collectively are responsible for ~8 million annual deaths. Particulate matter is the most dangerous form of air pollution, causing inflammatory and oxidative tissue damage. A deeper understanding of the physiological effects of particulate matter is needed for effective disease prevention and treatment. This review will summarize the impact of particulate matter on physiological systems, and where possible will refer to apposite epidemiological and toxicological studies. By discussing a broad cross-section of available data, we hope this review appeals to a wide readership and provides some insight on the impacts of particulate matter on human health.

## Introduction

In preindustrial civilizations, domestic combustion of biomass was the primary source of hazardous anthropogenic air pollution; the lungs of exhumed Ancient Egyptian mummies have been found blackened by it (1, 2). In Airs, waters and places written around the 4th century BCE, Hippocrates considered the relationship between air quality and health, whilst in the 1st century AD, Seneca The Younger wrote about the health benefits of escaping Rome's “ruinous mess of steam and soot” (3, 4). In response to Queen Eleanor's objections to the “unendurable smoke” emitted from bituminous sea coal fires, English Parliament passed the Smoke Abatement Law in 1273 (5). Nonetheless, London's air quality remained poor for centuries and was made significantly worse by the 12-fold increase in coal consumption during the Industrial Revolution (6). In Bleak House, Charles Dickens described the environment as “…Smoke lowering down from chimney-pots, making a soft black drizzle, with flakes of soot in it as big as full-grown snow-flakes gone into mourning, one might imagine, for the death of the sun…” (7). As well as increased respiratory disease risk, air pollution in Victorian Britain served as a selection pressure for peppered moth pigmentation. Here, predators could easily distinguish light-colored wings set against surfaces blackened by soot, and so over time darker wings were selected for and became the most common phenotype (8). After World War II, mass-export of high-quality coal left Londoners with little choice but to burn low-quality, high-sulfur lignite. In December 1952, cold weather increased coal use and a patch of high air pressure prevented dissipation of the soot and sulfur dioxide filled smoke (9). Within 5 days, The Great Smog of London killed around 12,000 people and precipitated the implementation of the UK Clean Air Act 1956, which was shortly followed by similar legislation in Europe and North America. In 1950, Europe and the United States contributed 85% of global CO_2_ emissions and by 2000 this figure was closer to 50% (10). Throughout the second half of the 20th century and beyond, industrialization of the Global South resulted in East Asia and South-East Asia becoming major contributors to global emissions (10). Today, of the 10 countries with the highest pollution-associated death rates, two are in South-East Asia (Bangladesh and India), two are in Europe (Georgia and Bosnia and Herzegovina) and six are in Africa (Chad, Nigeria, Somalia, Sierra Leone, Cote d Ivoire and Central African Republic) (11). In these countries (and many others) morbidity and mortality are driven by high population densities, traffic emissions, coal-fueled power stations and domestic use of biomass (12). Global socioeconomic inequity is a critical driver of air pollution-associated deaths; low- and middle-income countries account for 91% of the 8 million annual air pollution-associated deaths (12). This review provides an overview of the multiple physiological effects of particulate matter (PM) air pollution, including those on the respiratory cardiovascular, metabolic, endocrine, neurological and reproductive systems. The authors' intent has been to summarize a combination of recent and significant work in this growing research field.

## Particulate Matter

**Figure 1:**
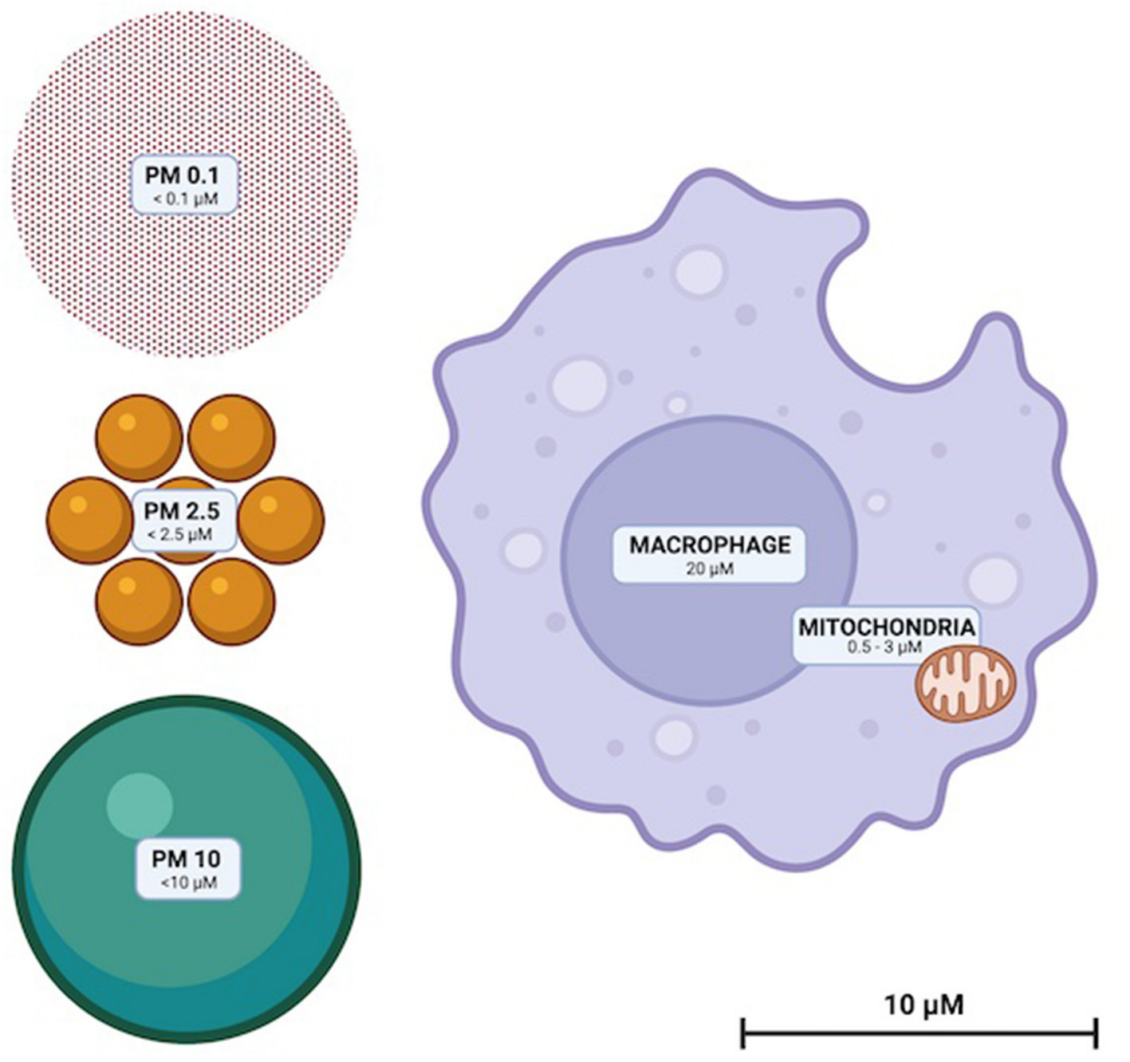
To scale illustration of the relative sizes of PM10, PM2.5, and PM0.1. Representative macrophage and mitochondria are included to scale for reference.

Particulate matter (PM) are solid compounds suspended in air that are sufficiently small to be inhaled (Figure 1). PM is categorized by particle diameter (measured in μm); PM0.1, PM2.5 and PM10 whilst ambient concentration is usually quantified as μg/m^3^. Some PM are of natural origin (bushfires, dust, sea spray, aerosols, etc.) but anthropogenic PM (diesel, coal and biomass combustion and emissions from metal refineries etc.) are the most dangerous to health (13). High atmospheric concentrations of human-made PM, and toxic and oxidative chemical characteristics render them disproportionately hazardous (13). Elemental and complex chemical species of PM are diverse, with surface shape, chemistry and charge impacted by emission source and environmental conditions. PM chemistry can change through reactions with other airborne PM and be affected by the oxidative effects of ozone and low ambient pH (14, 15).

**Figure 2:**
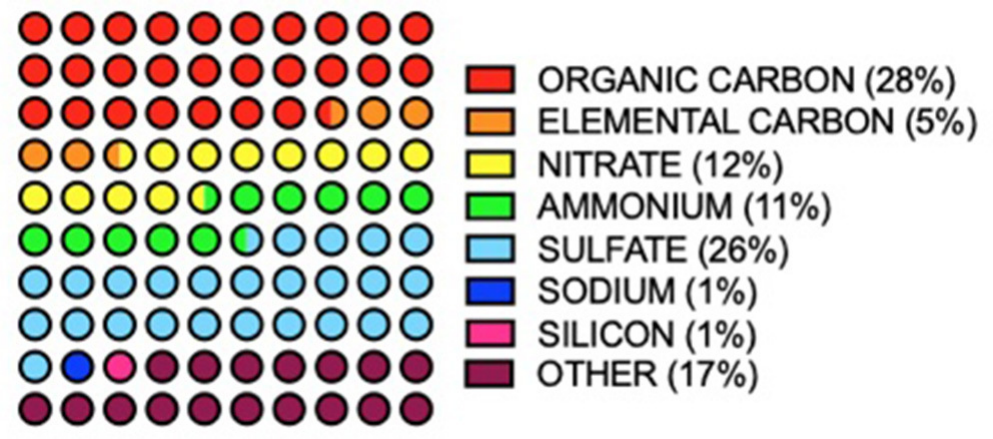
Annual averages of major PM2.5 components (>1 total mass%) in 187 USA counties between 2000 and 2005. Values from (16).

In air sampled from 187 counties in the USA between 2000 and 2005, 52 PM2.5 species were identified (16). Of this number, only seven species made up 83% of total PM mass (Figure 2). PM composition was found to be 28% organic carbon, 26%, sulfate, 12% nitrate, 11% ammonia, 5% elemental carbon, 1% silicon and 1% sodium (16). Components of the remaining 17% included alkali metals (K, Cs & Rb), alkaline earth metals (Ca, B, Mg & Sr), transition metals (Fe, Zn, Hf, Ta, V, Cd, Ti, Ag, Cu, Ir, M, Mn, Au, Mg, Hg, W, Sc, Cr, Nb, Zr, Ni, Y, and Co) basic metals, (Al, Sn, In, Pb, and Ga) semimetals (Sb), non-metals (P and Se), halogens (Cl and Br) and lanthanides (Ce, La, Eu, Tb, Sm and As) (16).

## PM Exposure

**Figure 3:**
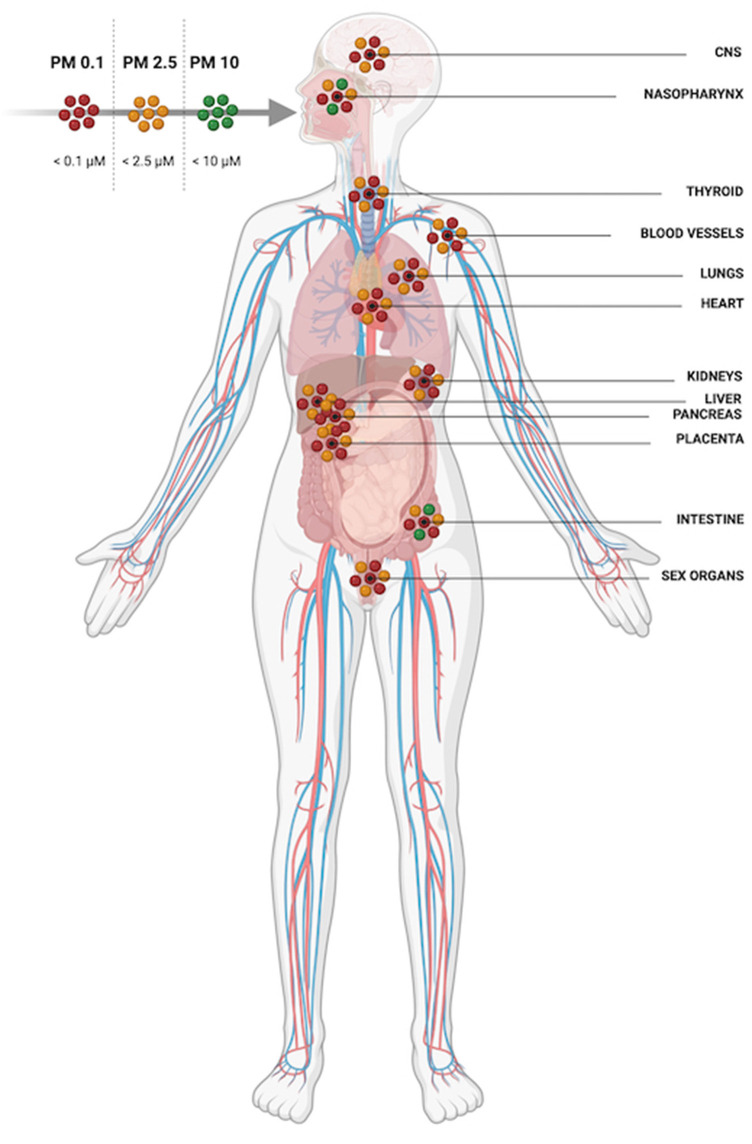
PM 10 is restricted to the upper airway and digestive system whilst PM2.5 and PM0.1 can be inhaled deeply into the lungs, translocate epithelial barriers and gain access to multiple organ systems.

When inhaled, larger diameter PM (>PM10) is limited to the upper airway whilst smaller particulates (< PM2.5) can access alveoli (Figure 3). PM2.5 can also cross respiratory endothelium, enter capillaries and systemic circulation thereafter (17). From the blood, PM2.5 can once again translocate endothelia once more and get into multiple extrapulmonary organs. In addition to inhalation exposure, particulates can enter circulation by crossing olfactory epithelia and *via* the gastrointestinal tract when swallowed after mucociliary removal from the lungs (18, 19). PM can damage endothelial cell layers as well as cross them; PM2.5 has been shown to compromise nasal epithelia intercellular tight junctions and reduce trans-epithelial resistance (20). A study in humans using gold nanoparticles as an inert proxy for PM demonstrated particles in the circulation as early as 6 h post inhalation (21). Nanoparticles were also detectable in the liver and in aortic atherosclerotic plaques and were detectable in participants' blood and urine for 3 months (21). In humans and animals PM has been found in multiple organs including the liver, kidneys and brain (17, 22–24).

## Disease Mechanisms

**Figure 4:**
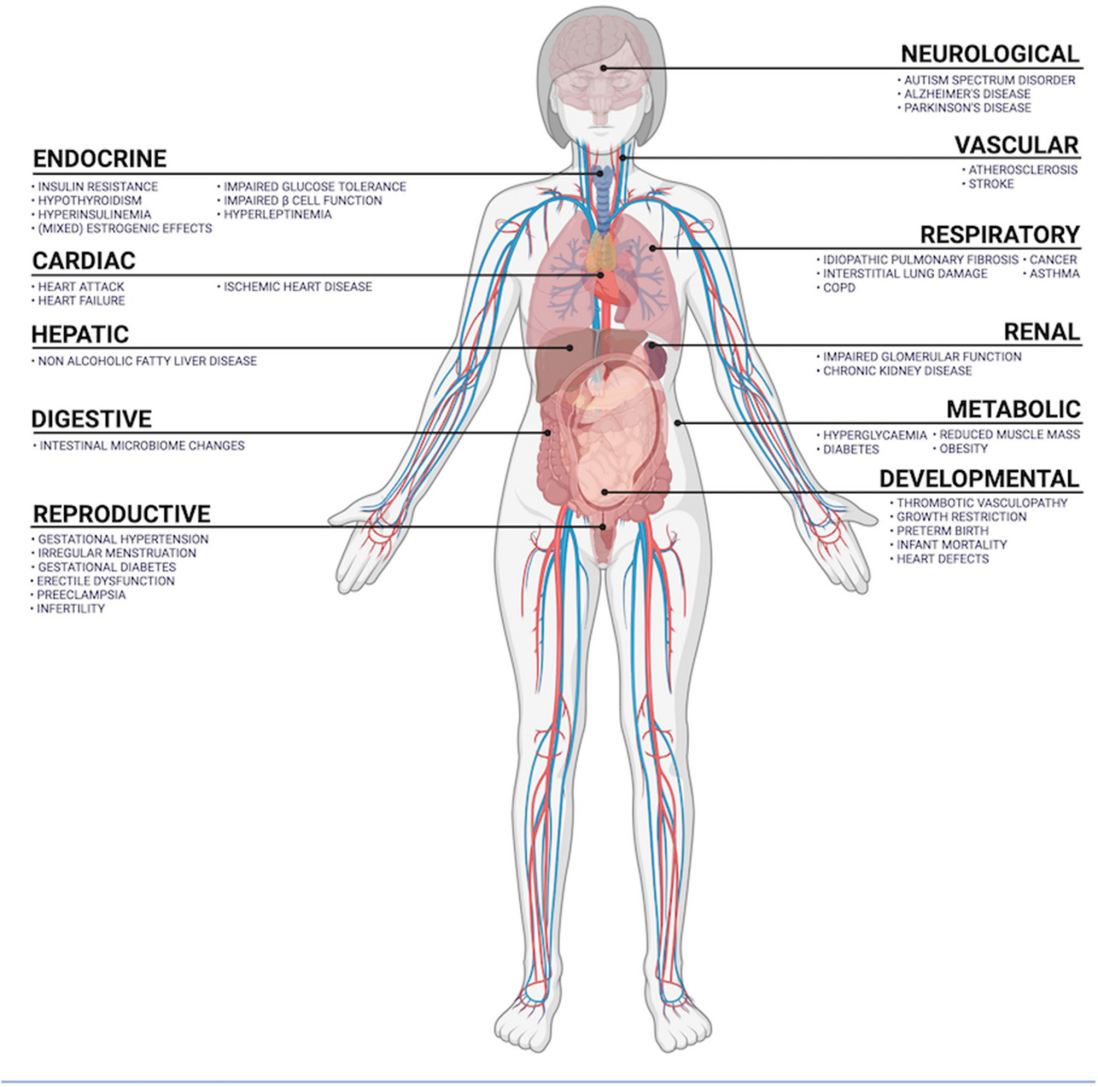
Summary of organ systems exposed to airborne PM and diseases positively correlated with PM exposure.

According to the World Health Organization, air pollution and climate change are the collective No. 1 threat to human health (25). Air pollution contributes to 9% of all global human deaths, and of these, 58% are from ischemic heart disease and cerebrovascular disease, 18% are from chronic obstructive pulmonary disease and acute lower respiratory tract infections, 6% are from lung cancer. Causes of death in the remaining 18% are mixed and many (12). Not all PM equally toxic, with the pathophysiological mechanisms varying between PM species (26). PM are mutagenic, can cause oxidative damage, activate inflammatory signal cascades and induce cell death (27–30). Toxicological research has investigated the differential oxidative and inflammatory effects of PM species (including black carbon, ammonia, nitrate and sulfate) and PM of varying origin (13). A common anthropogenic source of PM is incomplete combustion of diesel, gasoline, coal, and biomass (13). Trace metal content significantly contributes to the oxidative potential of PM (13, 26). The oxidative effects of PM can damage mitochondria, endoplasmic reticulum and DNA, can be carcinogenic and activate cell death signaling pathways (26). Inside cells, iron-based PM can overwhelm superoxide dismutase and glutathione peroxidase activity, inducing ferroptosis (29). PM2.5 can activate cytokine-dependent autophagy pathways, signaling through toll-like receptors, the Janus kinase-signal transducer and activator of transcription (JAK-STAT) pathway, and *via* cyclooxygenase 2-mitochondrial and prostaglandin E synthase. Here, increased tissue levels of C-reactive protein, tumor necrosis factor-α, interleukins 1, 6 and 8 (31–34). Inflammatory, oxidative, and toxic mechanisms are the primary effectors of PM-induced cell damage. Tissue-specific pathophysiology is an important determining factor in health outcomes, is discussed below and outlined in Figure 4.

## Respiratory Disease

The lungs are the primary site of PM-induced pathophysiology and best characterized in terms of the effects of PM exposure. Each 10 μg/m^3^ increase in ambient PM10 has been linked to a 0.58% increase in respiratory mortality, whist the same increase of PM2.5 has been associated with a 2.07% increase in respiratory disease hospitalization (35, 36). Research has shown human exposure to PM to be associated with multiple respiratory diseases including chronic obstructive pulmonary disease, asthma, interstitial lung damage and lung cancers (37–39). For patients with idiopathic pulmonary fibrosis, PM exposure has been shown to correlate with reduced lung forced vital capacity (39).

*Ex vivo* analysis of mouse lungs exposed to PM2.5 for three months exhibited significantly elevated levels of PM2.5, carbon monoxide, nitrogen oxides, interleukin-4, tumor necrosis factor-α and transforming growth factor-β1 when compared to controls (40). Of these circulating factors, interleukin-4 is known to promote B lymphocyte production of immunoglobulin E; a driver of allergic diseases including asthma and chronic obstructive pulmonary disease (41). PM2.5 exposure has been shown to induce pulmonary fibrosis both *in vivo* and *in vitro* experiments (42). PM2.5 increased tissue concentrations of transforming growth factor-β1; a fibroblast chemokine that can decrease protease secretion and increase extracellular expression of collagen and fibronectin (43). In a mouse model of idiopathic pulmonary fibrosis, *ex vivo* histological analysis revealed that exposure to black carbon PM2.5 aggravated lung inflammation and exacerbated histopathological changes to lung tissue including increased inflammatory cell infiltration and epithelial cell hyperplasia (44). This study also found that exposure to black carbon PM2.5 exacerbated already elevated interleukin-6 mRNA and reduced interferon-γ mRNA expression (44). Together, these preclinical studies not only highlight the potential danger of PM to health but also suggest that PM can increase the severity of existing health conditions like idiopathic pulmonary fibrosis. Increased counts of neutrophils, lymphocytes, eosinophils, M1 and M2 macrophages have been found in PM-exposed lung tissue (40). Whilst M1 macrophages can induce oxidative damage to lung epithelia, chronic elevation of M2 macrophages can cause pulmonary fibrosis and lung cancer (45–47). Mechanisms by which PM may induce fibrosis include increased intracellular edema, microvilli density, lamellar bodies and the density of macrophages containing endocytosed PM (40). In mice, PM2.5 exposure reduced mitochondrial density, increased NADPH oxidase 2 expression, significantly reduced total lung capacity, inspiratory capacity, and lung compliance (48). This same study found that PM2.5 exposure increased lung epithelia expression of N-Cadherin and reduced that of E-Cadherin; markers of epithelial-mesenchymal transition, a process common to cancer metastasis (48).

## Cardiovascular Disease

Air pollution is associated with elevated cardiovascular disease risk and cardiovascular disease-related mortality (49). PM2.5 exposure is linked to higher risk of heart attack, heart failure, ischemic heart disease, stroke, atherosclerosis, arrhythmia, hypertension, preeclampsia and neonatal hypertension (50–52). Air pollution exacerbates cardiovascular mortality risk for people with pre-existing cardiopulmonary disease (49). In adults, exposure to PM exposure has been linked to elevated systolic blood pressure and elevated pulse pressure, whilst in children, it has been found to associate with increased mean pulmonary arterial pressure and increased plasma endothelin-1 concentration (53, 54). Endothelin-1 is an endogenous atherogenic vasoconstrictor and may contribute to PM induced atherosclerotic plaque accumulation (55). The Multi Ethnic Study of Atherosclerosis (MESA) found short-term PM2.5 exposure to associate with to decreased flow-mediated vasodilation and vasoconstriction, indicating that particulates may impair endothelial function (56). Analysis of the same MESA cohort revealed a correlation between exposure to black carbon PM and pulmonary vascular remodeling (57). Here, changes in vascular volume – indicative of elevated blood pressure – were comparable to the effect of >15 pack years of cigarette smoking (57). PM exposure has been found to exacerbate high-risk atherosclerotic plaque progression, plaque destabilization and coronary calcification (58). PM exposure has also been linked to atrial fibrillation and reduced heart rate variability, with the later exacerbated by pre-existing diabetes (59). Preclinical models have demonstrated that PM can induce hypertension in healthy animals, secondary disease in animal models of heart failure and hypertension, and induce symptoms of cardiovascular dysfunction *via* central and renal cardiovascular regulation disruption (60–63). Exposure of rats to black carbon PM for 4 weeks dose and time dependently increased blood pressure (60). Four-day PM2.5 exposure to spontaneously hypertensive rats significantly increased heart rate and blood pressure and reduced heart rate variability (61). In a mouse model of chronic left ventricular heart failure, PM2.5 exposure significantly exacerbated lung oxidative stress, lung fibrosis, inflammation, vascular remodeling, and right ventricle hypertrophy (62, 63). Hypertensive, angiotensin II-infused apoe^−/−^ mice, exposed to PM2.5 for 4 weeks had a significantly increased incidence of abdominal aortic aneurysm compared to controls (64). Here, aortic aneurysm was associated with significant vascular elastin degradation, increased maximal abdominal aortic diameter and elevated expression of senescence proteins P16 and P21 (64). Increased vascular P21 is implicated in the development of atherosclerosis, causes of which include inflammation, hemodynamic damage and aberrant lipid metabolism (65, 66). Multiple models have shown PM2.5 exposure to stimulate endothelial release of inflammatory cytokines and adhesion molecules, promote macrophage infiltration, vascular smooth muscle cell dysfunction and plaque formation (67). Hypertensive, apolipoprotein-deficient mice exposed to PM2.5 for 3 months exhibited increased atherosclerotic lesion area, hepcidin and iron plaque depositions, increased plasma iron, ferritin, total cholesterol, low density cholesterol, vascular endothelial derived growth factor, monocyte chemoattractant protein-1 and pro-atherosclerotic cytokines interleukin 6 and tumor necrosis factor-α (64). Both blood pressure and heart rate are partially regulated by the central nervous system, with sympathetic output from the hypothalamus significantly impacting cardiovascular tone (68). In wild-type mice, long-term PM2.5 exposure has been found to increase basal blood pressure; an effect that was reversed with central alpha-2 adrenergic receptor antagonism. Concurrent inflammation of the hypothalamic arcuate nucleus was observed in hypertensive PM2.5-exposed mice (69). Increased noradrenergic signaling in the hypothalamic periventricular nucleus is known to increase sympathetic output and cardiovascular tone (70). Exposure of lean Brown Norway rats to PM for 1 day increased noradrenaline concentrations in the paraventricular nucleus and corticotropin releasing hormone concentration in the median eminence (71).

## Renal Disease

Human kidneys filter ~180 L of blood each day and are therefore vulnerable to PM exposure (21, 72). In people, PM2.5 exposure has been linked to an accelerated decline in glomerular filtration rate, diminished glomerular function during pregnancy, increased risks of chronic kidney disease, end stage renal disease, renal failure and chronic kidney disease mortality (72–74). Human studies have also revealed that PM2.5 exposure to positively correlate with risk of albuminuria; a marker of glomerular disfunction (72). A comparison of renal biomarkers in welders and office workers revealed that welders - exposed to much higher levels of PM2.5 that office worker controls – had elevated plasma markers of renal tubule damage; urinary kidney injury molecule-1 and neutrophil gelatinase-associated lipocalin (75). Another study investigating the potential for tubule damage in humans revealed a 10 μg/m^3^ increase in PM2.5 exposure to be associated with increased nephritis hospital admissions (76). PM2.5 exposure is associated with an elevated risk of adverse post kidney transplant outcomes, including acute rejection, graft failure and death (73, 74). A study specifically investigating the impact of PM on post-transplant outcomes found a 10 μg/m^3^ increase of PM2.5 to correlate with a 1.31-fold increase in the odds of transplant failure, a 1.59-fold increase in odds of delayed graft function and a 1.15-fold increase in all-cause mortality within 1 year of surgery (77). A similar study revealed an increase of 1 μg/m^3^ in PM10 exposure to be associated with increased risk of biopsy proven rejection, graft failure and mortality (78). Intratracheal exposure of PM2.5 to immunodeficient mice revealed no obvious renal histopathology. However, PM exposure was associated with elevated serum markers of renal damage including kidney injury molecule-1, cystatin C and uric acid. Moreover, 14-day PM exposure progressively increased renal concentrations of malondialdehyde, hydrogen peroxide, glutathione peroxidase, nuclear factor kappa-β, tumor necrosis factor-α, transcription factor protein-65, NADPH oxidase 4 and heme oxygenase-1 (79). In rats, sub-chronic exposure of PM2.5 resulted in elevated plasma β-2-microglobulin and cystatin-C; serum markers of early-stage kidney damage (80–82). PM exposure has also been found to induce histopathological lung damage, increase median blood pressure, increase urine volume and water consumption (80–82). Exposure of rats to diesel emission PM significantly reduced renal blood flow in controls and to a greater extent in rats with adenine-induced chronic kidney disease (81). Similar work in a mouse model of adenine-induced CKD revealed that PM exposure elevated renal tumor necrosis factor-α, lipid peroxidation, reactive oxygen species, collagen deposition, necrotic cell counts, dilated tubules cast formation and collapsing glomeruli (49).

## Endocrine Disease

The known effects of cigarette smoke on reproductive and thyroid hormones provide indications of the risks associated with PM exposure. Cigarette smoke is a risk factor in Graves hypothyroidism and is associated with elevated plasma cortisol, aldosterone, adrenal androgens and impacts female fertility by increasing steroid hormone binding globulin and decreasing circulating free estrogens (83–86). Several PM species have been identified as endocrine disrupting chemicals (87). In humans PM exposure is linked to insulin resistance, elevated circulating adipokines, hypothyroidism and (mixed) estrogenic effects (88). Thyroid hormones triiodothyronine (T3) and thyroxine (T4) regulate metabolic rate, cardiovascular tone and promote growth rate during fetal development and early life (89). In humans, PM exposure is associated with decreased plasma T4 both in pregnant women and new-borns, as well as congenital hypothyroidism and reduced infant birth weight (90). Black carbon, ammonia, organic matter and nitrate PM species appear to have the strongest links to thyroid dysfunction (91–94). Effective insulin signaling is required for glucose homeostasis, and insulin resistance is closely associated with obesity and is a risk factor for the onset of type-2 diabetes (95). PM exposure is associated with insulin resistance and non-alcoholic fatty liver disease, driven by oxidative stress and dyslipidaemia (96, 97). Together these studies highlight the link between air pollution and metabolic diseases including diabetes. Of >106 chemicals to which gas and oil extraction workers are exposed, 21 have been shown to exert estrogenic, androgenic and/or steroidogenic effects (98). Some chemicals identified as impacting endocrine function include benzene, toluene, ethylbenzene xylene, mercury, polychlorinated dibenzodioxins (PBDDs) and several polycyclic aromatic hydrocarbons (PAH) (88, 98, 99). Atmospheric sources of PAHs are vehicle emissions and biomass and coal combustion. Low molecular weight PAHs are in gas phase whereas high molecular weight PAHs are bound to the surface of PM (100). PAHs are classed as endocrine disrupting compounds and have been found to both increase and decrease estrogen receptor mRNA expression and function (REF). Estrogenic dysfunction has been shown to be both direct at estrogen receptors and indirect *via* aryl hydrocarbon receptor (AhR) signaling (101, 102). PBDDs also exert endocrine effects *via* AhRs, and preclinical experiments have shown AhR-mediated effects of dioxin exposure to include weight loss, reproductive and developmental toxicity, tumorigenesis and immune system dysfunction (103). PM contains many metal elements, some of which interfere with estrogenic signaling by mimicking endogenous estrogens (104). Metalloestrogens include aluminum, selenium, antimony, arsenic (arsenite; NaAsO_2_), barium, cadmium, chromium, cobalt, copper, lead, mercury, nickel, tin and vanadium (vanadate; V_2_O_5_) (16, 104).

## Obesity and Diabetes

In humans, the association between PM2.5 exposure and obesity is dependent on age, gender and socioeconomic demographic (105, 106). A growing body of evidence indicates that PM2.5 exposure is a risk factor for reduced skeletal muscle mass, obesity, diabetes and hypertension (107–109). Long-term PM exposure is associated with a high risk for type 2 diabetes, and road traffic-specific PM is correlated with an elevated risk (110). Increased incidence of type-2 diabetes remains when adjusted for age, body mass index (BMI), and socioeconomic status (111, 112). PM exposure is associated with higher levels of circulating complement factor 3 (C3c), and women with elevated plasma C3c are more susceptible to diabetes than those with low C3c (112). PM2.5 exposure is associated with a faster decline in insulin sensitivity during childhood and higher BMI by age 18 (113–115). The associated between PM exposure and hypertension is greater in overweight and obese children (116). In animal studies, exposure of rats to PM increased chocolate consumption whereas in chow-fed wild-type mice, 10-week PM2.5 exposure increased visceral fat mass, insulin resistance and adipose tissue inflammation (117, 118). In mice, short-term PM exposure increased food intake, fat mass and UCP-1 expression in brown adipose tissue (119). PM exposure also induced hypothalamic inflammation indicated by increased microglia density, increased toll-like receptor-4 and elevated inhibitory nuclear factor-kappa-B-kinase-epsilon expression (119). After 12 weeks of PM exposure, mice exhibited increased food intake and elevated fat mass and had lower energy expenditure. Mice had elevated levels of plasma leptin and insulin and increased Homeostatic Model Assessment for Insulin Resistance (HOMA-IR) indicators of insulin resistance (119). This same study also revealed that PM exposure decreased hypothalamic satiety markers, including reduced levels of phosphorylated STAT 3, and diminished proopiomelanocortin expression (119).

PM exposure to mice was found to induce hepatic oxidative stress, inflammation, negatively affect glucose tolerance and induce insulin resistance (96, 120). Interestingly PM exposure has been found to increase hepatic triacylglycerols, free fatty acids and cholesterol levels in female but not in male mice (96). In addition to insulin resistance, PM exposure has been shown to exert toxic effects directly on the pancreas (121). In a streptozotocin-induced mouse model of type-1 diabetes, PM from diesel exhaust fumes exacerbated pancreatic cell vacuolation and islet cell apoptosis, increased pancreatic amylase activity, increased expression of oxidative stress markers 8-isoprostane and superoxide dismutase and reduced levels of the antioxidant glutathione peroxidase (121). In a rat model of gestational diabetes PM exposure induced maternal pancreatic inflammation indicated by diminished pancreatic glucose transporter-2 expression (122).

## Gastrointestinal Disease

Mucociliary clearance of PM from the lungs followed by its ingestion within saliva leads to gastrointestinal PM exposure. A growing body of preclinical data has revealed PM-induced gastrointestinal inflammation and gut microbiome changes (123–125). In mice, PM exposure altered the relative proportions of microbiota component species, impaired gut permeability through oxidative stress and increased proinflammatory cytokine expression in an interleukin-10 knock out model of inflammatory bowel disease (123–125). However, epidemiological studies have not yet identified a clear link between PM exposure and inflammatory bowel disease (126).

## Neurological Disease

Increased ambient PM concentration positively correlates with the incidence of Alzheimer's disease, Parkinson's disease, Multiple Sclerosis, dementia and autism spectrum disorder (Figure 2) (127). Long-term PM2.5 exposure significantly increased age adjusted risk of mortality and hospital admission for Alzheimer's disease, Parkinson's disease and non-Alzheimer's disease dementia (128). This study found the strongest correlation to exist between PM2.5 and Alzheimer's disease (128). One longitudinal study found that people living within 50 meters of a main road had a 12% greater chance of dementia diagnosis (129). PM2.5 exposure is linked to faster decline in new learning and immediate recall, as well as MRI-detected gray matter atrophy in brain areas vulnerable to Alzheimer's disease pathology (130). PM2.5 exposure has been linked to Alzheimer's specific cognitive impairments (CERAD score but not ABC score) however post-mortem analysis of neuropathology in the brains of Alzheimer's disease patients failed to reveal any link between PM2.5 exposure 10 years before death, and disease progression indicated by Braak stage (131). The impact of specific PM (including black carbon, organic matter, nitrate, sulfate, sea salt and soil) exposure on the rate of initial Parkinson's disease hospitalization in New York State was investigated. This study revealed that with each standard deviation increase in either nitrate or organic matter PM, the risk of hospitalizations increased 1.06-fold (132).

PM 0.1 can cross the blood brain barrier and cause inflammatory and oxidative tissue damage as well as microglial activation (133). Glutamatergic excitotoxicity is a common reported endpoint for acute PM induced pathophysiology in the central nervous system. PM has been found in neurons, glia, endothelium, choroid plexus ependymal cells, cerebrospinal fluid, nasal epithelium, and olfactory epithelium of individuals subjected to PM exposure (134). PM2.5 has been found to reduce nervous system expression of the tight junction proteins, zonula occludens 1 and 2 (135). This study found a compromised blood brain barrier permeable to macrophage infiltration, and nervous system tissue subject to glutamatergic excitotoxicity, triggered by macrophage-derived glutamate (135). In mice, PM2.5 has been shown to reach the olfactory bulb and induce microglial activation and glutamatergic excitotoxicity that could be blocked with the antioxidant *N*-acetylcysteine (136).

Alzheimer's disease is characterized by cortical and hippocampal amyloid-β plaque and tau tangle deposition. Amyloid-β plaque formation and gliosis underlie at least some of the cognitive deficits associated with AD progression (137). In a transgenic mouse model of Alzheimer's disease, exposure to diesel emission PM2.5 exacerbated amyloid-β plaque deposition, and increased astrocytosis and microgliosis. Additionally, elevated inflammatory cytokines including tumor necrosis factor, nuclear factor-α, interleukins 1β and 6, interferon-γ and macrophage inflammatory protein-3α were identified in the cortices of double transgenic mice (138). In a similar study, 13-week exposure to diesel exhaust PM also accelerated cortical amyloid-β plaque deposition, an effect associated with significant impairments to motor coordination (139). Parkinson's disease is caused by loss of dopaminergic neurons in the substantia nigra of the basal ganglia. Neuron loss results in diminished cortical input and associated behavioral and cognitive deficits. In a rotenone-induced mouse model of Parkinson's disease, PM2.5 exposure induced mitochondrial dysfunction, oxidative stress and apoptosis in the substantia nigra. In the same study, PM exposure also exacerbated motor and somatosensory deficits (140). Multiple Sclerosis (MS) is a progressive, demyelinating and neurodegenerative disease of the CNS. Short-term PM exposure is associated with increased MS hospital admissions and relapse (127). In a mouse model of lipophosphatidylcholine-induced demyelination, PM exposure impairs myelin repair and sustains astroglia and microglia dependent neuroinflammation. PM2.5 exposure to rats impaired spatial learning and memory, inquiring ability and sensory function, these changes were related to ultrastructural changes to mitochondria and myelin (141). Mice exposed to PM2.5 for 10 months developed structural hippocampal alterations including diminished apical spine density and dendritic branching of hippocampal neurons and behavioral studies revealed reduced spatial learning and memory impairments (142).

## Developmental and Gestational Disease

PM2.5 has been found on the fetal side of the placenta. Given the ability of PM to cross endothelial barriers, it is possible that during pregnancy, PM impacts gestation at the level of the mother, the fetus and the placenta. The placenta is critical to fetal development and dysfunction can lead to preeclampsia, gestational diabetes, fetal growth restriction fetal thrombotic vasculopathy, congenital heart defects, reduced birth size, birth weight, preterm birth, and infant mortality (143–151). Pathophysiological mechanisms of PM-induced placental damage may include oxidative stress, inflammation, coagulation and endothelial dysfunction (152).

In rodents, PM exposure at later gestational stages has been shown to decrease gestational duration and birth weight (153). A similar study also found that this lower body weight was exacerbated during lactation. Here, pups exposed to PM *in utero* were even lighter relative to controls by time of weaning (154). In rats, PM exposure increased blastocyst absorption, reduced maternal weight gain and fetal weight (155). PM2.5 exposure to pregnant Sprague Dawley rats caused increased blood pressure of pups as well as reduced sodium excretion, reduced renal dopamine 1 receptor expression and dopamine 1 receptor-mediated natriuresis and diuresis (156). The placentas of mice exposed to PM during gestation, exhibited increased inflammation and embolism, furthermore maternal blood contained elevated circulatory mononuclear cells, platelets and levels of interleukin-6 (155). At the embryonic level, trophoblasts have been shown to endocytose PM and when exposed to PM, human trophoblasts have been found to exhibit reduced cell growth, endoplasmic reticulum stress and decreased beta-human chorionic gonadotropin secretion (157).

## Perspective and Conclusions

It is well documented that PM is associated with harmful outcomes to animal and human health. In addition to direct exposure of the respiratory system, airborne particulates can cross endothelial barriers, enter circulation, and accumulate in multiple organ systems. As we integrate current environmental changes in our research a rigorous multidisciplinary approach is necessary to ascertain the extent to which individual and relevant combinations of PM impact human health. Understanding the exact inflammatory, toxic and oxidative mechanisms of pathophysiology in all exposed physiological systems is needed in order to improve global health outcomes. More preclinical research into the respiratory, cardiovascular, endocrine, metabolic, digestive, reproductive and neurological effects of PM exposure is required to inform prevention, treatment, and policy change. Future work in the field of pollution and physiology should determine the extent of damage, mechanisms of pathophysiology, time course and reversibility of PM induced health outcomes. Due to the ubiquity of PM in the organs of exposed subjects, the extent of required research is vast, but will undoubtedly expedite improved methodologies of prevention and treatment for PM associated diseases.

## Author Contributions

Manuscript written by JP and SS. Manuscript edited by JP, LC, and SS. All authors contributed to the article and approved the submitted version.

## Funding

SS has been supported by both NHMRC and NHF, Australia.

## Conflict of Interest

JP was employed by Woodrudge LTD. The remaining authors declare that the research was conducted in the absence of any commercial or financial relationships that could be construed as a potential conflict of interest.

## Publisher's Note

All claims expressed in this article are solely those of the authors and do not necessarily represent those of their affiliated organizations, or those of the publisher, the editors and the reviewers. Any product that may be evaluated in this article, or claim that may be made by its manufacturer, is not guaranteed or endorsed by the publisher.
